# Estimation of Geographic Variation in Human Papillomavirus Vaccine Uptake in Men and Women: An Online Survey Using Facebook Recruitment

**DOI:** 10.2196/jmir.3506

**Published:** 2014-09-01

**Authors:** Erik J Nelson, John Hughes, J Michael Oakes, James S Pankow, Shalini L Kulasingam

**Affiliations:** ^1^School of Public HealthDivision of Epidemiology and Community HealthUniversity of MinnesotaMinneapolis, MNUnited States; ^2^School of Public HealthDivision of BiostatisticsUniversity of MinnesotaMinneapolis, MNUnited States

**Keywords:** online recruitment, social media, Facebook, local estimation, geographic variability, human papillomavirus, HPV

## Abstract

**Background:**

Federally funded surveys of human papillomavirus (HPV) vaccine uptake are important for pinpointing geographically based health disparities. Although national and state level data are available, local (ie, county and postal code level) data are not due to small sample sizes, confidentiality concerns, and cost. Local level HPV vaccine uptake data may be feasible to obtain by targeting specific geographic areas through social media advertising and recruitment strategies, in combination with online surveys.

**Objective:**

Our goal was to use Facebook-based recruitment and online surveys to estimate local variation in HPV vaccine uptake among young men and women in Minnesota.

**Methods:**

From November 2012 to January 2013, men and women were recruited via a targeted Facebook advertisement campaign to complete an online survey about HPV vaccination practices. The Facebook advertisements were targeted to recruit men and women by location (25 mile radius of Minneapolis, Minnesota, United States), age (18-30 years), and language (English).

**Results:**

Of the 2079 men and women who responded to the Facebook advertisements and visited the study website, 1003 (48.2%) enrolled in the study and completed the survey. The average advertising cost per completed survey was US $1.36. Among those who reported their postal code, 90.6% (881/972) of the participants lived within the previously defined geographic study area. Receipt of 1 dose or more of HPV vaccine was reported by 65.6% women (351/535), and 13.0% (45/347) of men. These results differ from previously reported Minnesota state level estimates (53.8% for young women and 20.8% for young men) and from national estimates (34.5% for women and 2.3% for men).

**Conclusions:**

This study shows that recruiting a representative sample of young men and women based on county and postal code location to complete a survey on HPV vaccination uptake via the Internet is a cost-effective and feasible strategy. This study also highlights the need for local estimates to assess the variation in HPV vaccine uptake, as these estimates differ considerably from those obtained using survey data that are aggregated to the state or federal level.

## Introduction

Human papillomavirus (HPV) is the most common sexually transmitted infection in the United States [1] and is the necessary cause of cervical cancer [2]. HPV infections are also associated with other cancers (eg, anogenital and oropharyngeal) as well as genital warts [3,4]. In total, it is estimated that 5.2% of cancers in men and women worldwide are attributable to HPV [5].

Two vaccinations against HPV infection are currently licensed in the United States. The vaccinations were originally licensed for use in girls, but as of October 2011, the Advisory Committee on Immunization Practices extended their recommendation of the quadrivalent vaccine to include both boys and girls aged 11 or 12 years old [6,7]. However, vaccine uptake has been far lower than expected, with only about half of eligible young women receiving at least one dose of the vaccine [8]. Initiation of the HPV vaccine series has been shown to be higher among minority adolescent girls; however, completion of the three-dose series is substantially lower among black and Hispanic adolescent girls compared to white adolescent girls [9]. Although male vaccination data are very limited (due to a later date of approval of the HPV vaccine for boys), racial and income differences in terms of vaccine series initiation and completion have also been observed among adolescent boys [10].

Previous research on HPV vaccine coverage has used publicly available data from five national health surveys (National Survey of Family Growth, National Immunization Survey [NIS]-Teen, National Health and Nutrition Examination Survey, National Health Interview Survey [NHIS], and the Behavioral Risk Factor Surveillance System) [11-15]. These surveys are designed to gather information on a variety of health topics and ask only a few questions regarding HPV vaccination. However, none of these surveys address cervical cancer screening practices and potential barriers to screening or HPV vaccine receipt. In addition, due to the small number of responses in many geographic areas, local data from these surveys are routinely suppressed and aggregated to state boundaries in order to protect the confidentiality of survey respondents, which means that variations at a local level (ie, between counties or postal codes) cannot be adequately assessed. Further, these surveys have, to date, primarily surveyed adolescent girls; HPV vaccination practice data of adolescent boys are limited [8].

The Internet provides a unique point of contact to reach young adults for health research. Several studies have demonstrated that Internet-based research can be used to elicit high response rates at a fraction of the cost of traditional recruitment methods [16-18]. In addition, it has been shown that when compared to in-person interviews, Internet-based surveys have the potential to reach more respondents, include otherwise inaccessible populations, and reduce bias in responses as respondents may be willing to report more sensitive information online compared to in-person interviews [19-24]. A number of studies have also shown that recruitment via Facebook (the leading social media site with more than one billion active users worldwide) can be used to enroll representative samples of the general population [16,25-30]. This combination of reach, utility, and reduced cost indicates that social media networks can be a cost-effective medium for research.

The objective of this study was to estimate HPV vaccination practices among a local population of young adult men and women in the United States using an Internet-based recruitment strategy.

## Methods

### Participants

Men and women from Minnesota were surveyed about their HPV vaccination practices via the Internet from November 21, 2012, through January 31, 2013. Participants were English-speaking, aged 18-30 years, had a Facebook account, and resided in the greater Twin Cities Metropolitan Area (ie, within 25 miles of downtown Minneapolis, MN). This age range was used to target men and women who were eligible to receive the HPV vaccine, participate in cervical cancer screening (women), and able to provide informed consent. The Twin Cities Metropolitan Area was selected due to the variation of HPV-related cancer incidence rates exhibited in this area during the past 15 years, the high concentration of colleges and universities, and the large population of 18-30 year olds residing in this area [31]. The University of Minnesota Institutional Review Board approved this study.

### Facebook Recruitment Campaign

**Figure 1:**
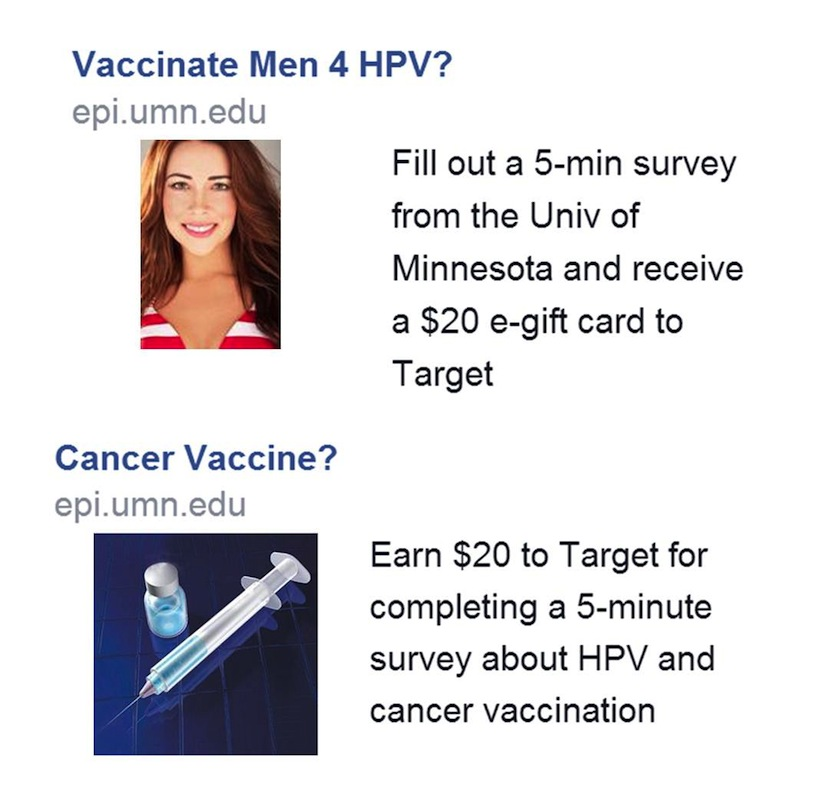
Examples of Facebook advertisements.

Participants were recruited online via Facebook advertisements (Figure 1). Tailored advertisements were used to target Facebook users who had profiles that matched the study inclusion criteria. The advertisement criteria were adjusted as needed to target specific postal codes with fewer responses in order to achieve a balanced sample of participants by postal code. Facebook uses an advertisement algorithm that automatically selects the best advertisement to display based on its performance and the advertiser’s bid [32]; 14 unique advertisements were created and approved by Facebook. For this study, multiple advertisements were submitted for auction simultaneously to create a continuous recruitment window in the event that a particular advertisement performed poorly. Bidding prices and advertisement availability (advertisements can be paused and released at the discretion of the advertiser) were monitored daily and adjusted as necessary until the intended number of completed questionnaires was obtained. The bidding price for advertisements ranged from US $0.75 to US $2.75, with a maximum daily budget of US $50. When a Facebook user clicked on the study advertisement, they were automatically redirected to the secure study website and invited to complete a questionnaire regarding HPV vaccination practices.

The Facebook Ads Manager was used to track the total number of impressions (each time an advertisement was displayed), the number of times an ad was clicked, the average cost-per-click, and the number of people reached (ie, the number of Facebook users that had an opportunity to view one of the study advertisements). Google Analytics software was used to tabulate the total number of visits, the unique visits, the average duration of visits, and the bounce rate (the percentage of visitors that visit a website and leave the site without further browsing) of the study website.

### Study Procedures

Participants who clicked on a Facebook advertisement were directed to a secured study website and were provided with information regarding the purpose of our study. Participants provided informed consent by clicking on a button that directed them to the study questionnaire. After providing consent, study participants were immediately asked to self-report their age and state of residence. Participants who did not meet the age criteria or who reported that they did not live in Minnesota were considered ineligible and were disqualified from answering the remainder of the questionnaire. Study participants who met the eligibility criteria were also asked to self-report their gender, postal code of their home address, race/ethnicity, highest level of education attained, attendance at religious services, political preferences, sexual orientation, their awareness of HPV, and whether or not they had received the HPV vaccine. Conditional upon participants’ responses, skip logic patterns (ie, participants skip over survey questions that, based on their answers to other questions, do not need to be filled out) were implemented in order to ask applicable follow-up questions regarding the number of shots received, the vaccine type (quadrivalent/bivalent), and reason(s) for not having received the vaccination, as well as future vaccination intentions. Female participants were also asked a series of adaptive questions about past cervical cancer screening. The survey questions regarding HPV vaccination and cancer screening that we used in this study were questions used in the five national surveys mentioned above, in order to facilitate comparisons between studies. Participants were not required to answer every question and could exit the survey at any time. Computer Internet Protocol (IP) addresses were tracked, and multiple entries from the same IP address were not accepted. Survey responses that contained repeated email addresses across multiple survey attempts (n=86) were not accepted. Additionally, 8 surveys that were only partially completed (ie, the participant withdrew) were not included in the analyses. The survey was anonymous and was administered using the online survey assessment tool SurveyMonkey. Eligible respondents who provided informed consent and completed the online survey were emailed an electronic gift card in the amount of US $20 for Target.

## Results

**Figure 2:**
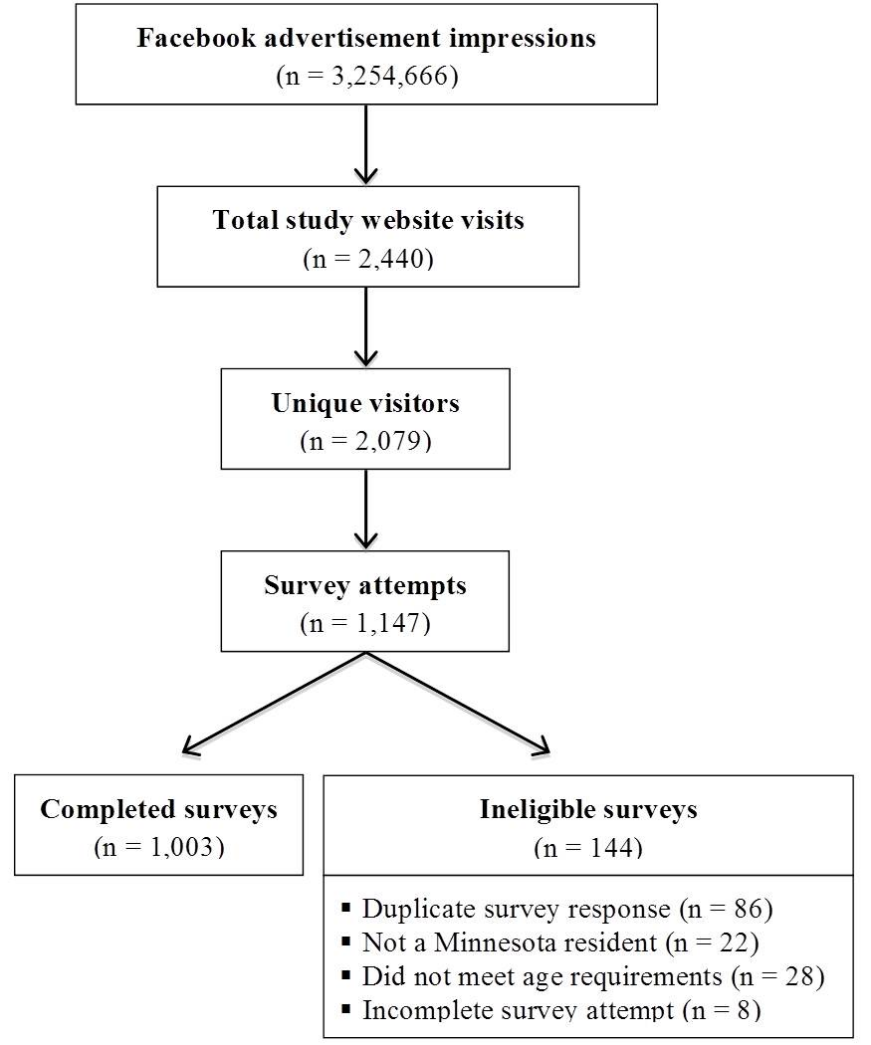
Recruitment summary flowchart.

**Figure 3:**
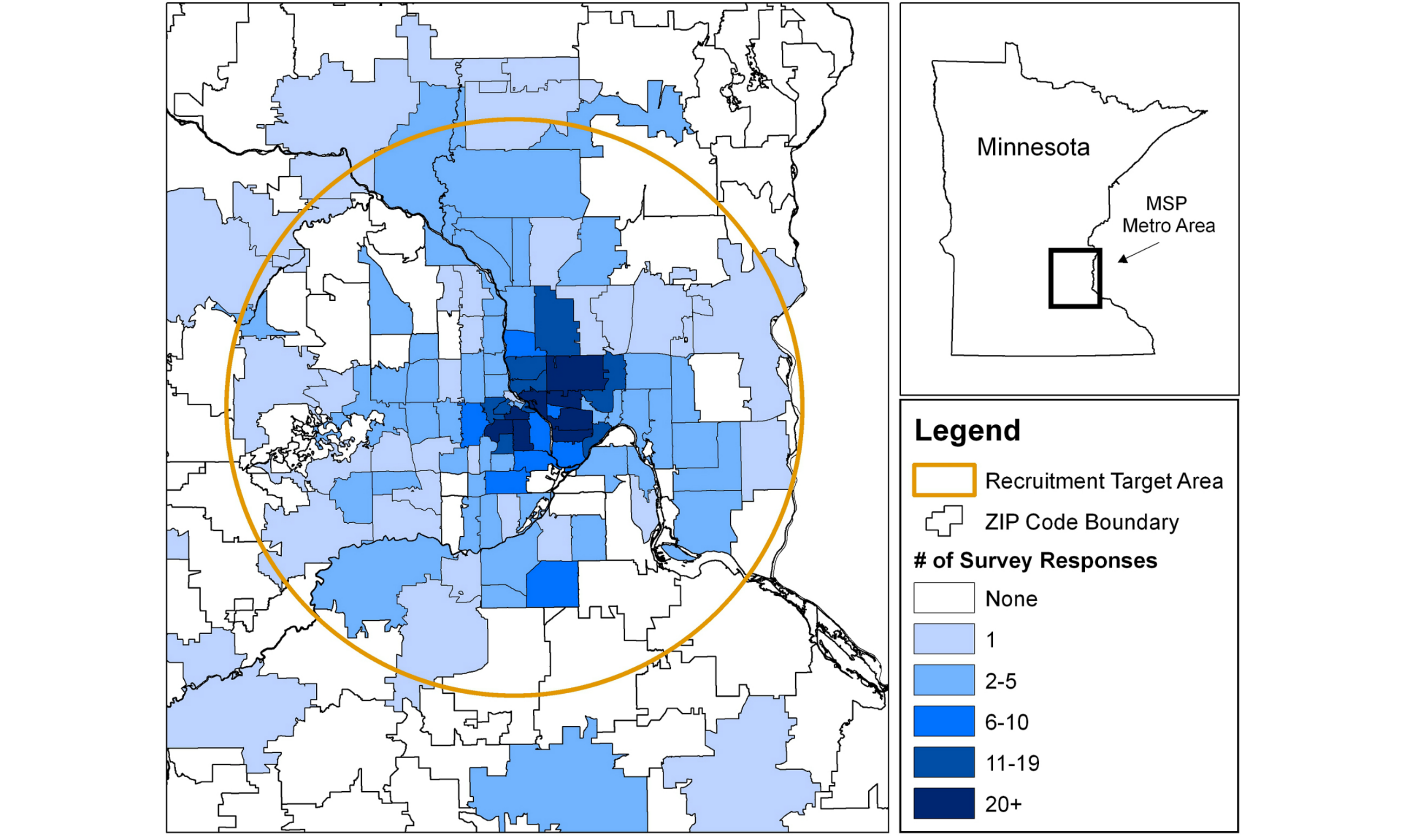
Map of the recruitment target area and the number of completed surveys by ZIP code.

Of the 2079 men and women who were recruited via Facebook and visited the study website, 1003 (48.24%) enrolled in the study and completed the survey. Targeted advertising within Facebook based on geographic and age criteria limited the number of ineligible participants (4.4% of all survey attempts) who attempted to access the survey. In total, 86 survey attempts (7.5% of all survey attempts) were identified as duplicate surveys, indicating that an individual attempted to complete the survey more than once (Figure 2). Facebook advertising and recruitment resulted in an average cost of US $1.36 per completed survey. In addition, 90.6% (881/972) of study participants who self-reported their postal code were located within the recruitment target area (ie, located within a 25-mile radius of downtown Minneapolis, Minnesota; Figure 3).

The recruitment target area for this study was a 25-mile radius from downtown Minneapolis, Minnesota. Of the 972 participants who reported their postal code, 881 (90.6%) lived within the recruitment study area.

A total of 1003 participants (557 women and 446 men) completed the online survey. Characteristics of the study population are presented in Table 1. With respect to race/ethnicity, the study population was broadly similar to that of 18-34 year-olds in the greater Minneapolis-St. Paul Metropolitan Area based on US Census data. However, due to the inclusion and exclusion criteria, the study population was more educated than the general population of 18-34 year-olds in the Minneapolis-St. Paul Metropolitan Area. In all, 44.2% of respondents (396/896) who knew of the HPV vaccine had been vaccinated against HPV (ie, received ≥1 dose of HPV vaccine), with 65.6% of women (351/535) having been vaccinated with ≥1 dose of HPV vaccine compared to 13.0% of men (45/347). Completion of the HPV vaccine series (ie, receipt of all 3 doses) was reported by 74.9% of women (263/351) and 22.2% of men (10/45) who had ever received an HPV vaccine (Table 2). Among the 351 women who had received ≥1 dose of HPV vaccine, 265 (75.5%) had also received at least one Pap smear in their lifetime. Of the 479 unvaccinated men and women, 403 (84.1%) were not interested or were unsure about receiving the vaccine in the future.

**Table 1:** Selected study participant characteristics compared to US Census estimates for Minneapolis and St. Paul, Minnesota.^a^

		Study participants			Census data
		Men, n=446	Women, n=557	Total, N=1003	Minneapolis/St. Paul, %
Mean age, years		23	23	23	18 to 34
**Race, n (%)**					
	White	384 (86.3)	457 (82.3)	841 (84.10)	79.3
	Black	17 (3.8)	33 (5.9)	50 (5.00)	9.1
	Asian	30 (6.7)	30 (5.4)	60 (6.00)	8.1
	American Indian or Alaska native	2 (0.4)	7 (1.3)	9 (0.90)	0.8
	Native Hawaiian or Pacific Islander	1 (0.2)	3 (0.5)	4 (0.40)	0.03
	Other	11 (2.5)	25 (4.5)	36 (3.60)	2.6
**Ethnicity, n (%)**					
	Hispanic	15 (3.4)	19 (3.4)	34 (3.42)	5.2
	Non-Hispanic	427 (96.6)	533 (96.6)	960 (96.58)	94.8
**Education, n (%)**					
	<High school	2 (0.4)	0 (0.0)	2 (0.20)	1.9
	Some high school	6 (1.3)	8 (1.4)	14 (1.40)	8.0
	High school graduate	36 (8.1)	36 (6.5)	72 (7.19)	21.9
	Some college/tech. school	190 (42.7)	209 (37.6)	399 (39.86)	36.3
	College graduate	167 (37.5)	237 (42.6)	404 (40.36)	25.1
	Graduate school	44 (9.9)	66 (11.9)	110 (10.99)	6.8

^a^Data are 5-year estimates for 18-34 year-olds in the Minneapolis-St. Paul Metropolitan Area as described in the 2006-2010 American Community Survey of the United States Census Bureau.

**Table 2:** Selected survey responses regarding vaccination against human papillomavirus.

Survey question		Men (n=446)	Women (n=557)	Total (N=1003)
		n (%)	n (%)	n (%)
**Ever heard of HPV** ^a^				
	Yes	409 (93.0)	536 (96.8)	945 (95.07)
	No	31 (7.0)	18 (3.2)	49 (4.93)
**Ever heard of HPV vaccine**				
	Yes	361 (82.4)	535 (96.6)	896 (90.32)
	No	77 (17.6)	19 (3.4)	96 (9.68)
**Ever had an HPV vaccination among those who had heard of the HPV vaccine**				
	Yes	45 (13.0)	351 (66.5)	396 (45.26)
	No	302 (87.0)	177 (33.5)	479 (54.74)
**Number of HPV shots received**				
	1 shot	11 (24.4)	31 (8.8)	42 (10.61)
	2 shots	14 (3.9)	38 (10.8)	52 (13.13)
	3 shots (complete vaccine series)	10 (22.2)	263 (74.9)	273 (68.94)
	Don’t know	10 (22.2)	19 (5.4)	29 (7.32)
**Likelihood of HPV vaccine receipt in the next 12 months among those not vaccinated** ^a^				
	Very likely	7 (2.3)	13 (7.3)	20 (4.18)
	Somewhat likely	26 (8.6)	30 (16.9)	56 (11.69)
	Not too likely	75 (24.8)	47 (26.6)	122 (25.47)
	Not likely at all	173 (57.3)	84 (47.5)	257 (53.65)
	Not sure/don’t know	21 (7.0)	3 (1.7)	24 (5.01)
**Reason stated for not receiving the HPV vaccine in the next 12 months** ^a^				
	Not needed or necessary	140 (52.4)	40 (31.3)	180 (45.57)
	Not sexually active	33 (12.4)	23 (18.0)	56 (14.18)
	Knowledge^b^	25 (9.4)	10 (7.8)	35 (8.86)
	Safety concerns/side effects	9 (3.4)	22 (17.2)	31 (7.85)
	Costs	13 (4.9)	14 (10.9)	27 (6.84)
	Already have HPV	19 (7.1)	5 (3.9)	24 (6.08)
	Monogamous	8 (3.0)	6 (4.7)	14 (3.54)
	Other^c^	6 (2.2)	6 (4.7)	12 (3.04)
	Not for men	11 (4.1)	0 (0.0)	11 (2.78)
	Too old	3 (1.1)	2 (1.6)	5 (1.27)

^a^Responses presented are for the 479 individuals who reported not having been vaccinated against HPV.

^b^Don’t know about HPV or HPV vaccine.

^c^Responses included “fear of needles”, “too busy/no time”, “don’t use vaccines”, or “already sexually active”.

## Discussion

### Principal Findings

In this study, we found that recruiting a locally representative sample of young adults via the Internet to participate in a survey about HPV vaccination was cost-effective and efficient. Approximately half of the 2079 individuals that clicked on an advertisement and visited our study website participated and completed our survey at an estimated advertising cost of US $1.36 per enrolled participant. Consistent with other studies, this study found that using the Internet, and in particular social media sites such as Facebook, is successful for recruiting and engaging young adults and hard-to-reach populations for health research [16-18]. This method of recruitment is particularly noteworthy given declining response rates from traditional recruitment techniques such as random digit dialing or mailed surveys [33-35]. In addition to higher participation rates, the targeted advertising features embedded within social media websites drastically reduce costs associated with identifying and reaching a large pool of eligible participants [25,26,28]. The targeted advertising used in this study also allowed us to collect data within an accelerated timeline (eg, pilot testing of a specific intervention) from a specific geographic location.

Notably, the characteristics of our study population were similar to those of the source population. An estimated 90% of Internet users aged 18-29 years in the United States access social media sites (71% accessed Facebook) in 2013; thus, this finding is likely attributable to the wide reach of social media recruitment [36]. However, our study population was more educated than the general population in the Minneapolis-St. Paul Metropolitan Area, which may be due to the large number of colleges and universities in this area. It cannot be ruled out that people with lower education were less likely to access Facebook and view the advertisements, although other studies have shown that lower income and less educated participants are as likely to participate in Internet-based research studies as those with higher incomes and higher levels of education [26,37,38].

In this study, we were also able to collect detailed HPV vaccination data, including participation in screening (for women) and potential barriers to receiving these services among a representative sample of men and women in a defined local geographic area. National surveys including the Behavioral Risk Factor Surveillance System, the NHIS, and the NIS-Teen do not simultaneously assess these factors within the same respondents in their populations. Additionally, these (and other) national surveys aggregate or suppress responses due to participant identification concerns and consequentially local variation and patterns may be obscured. However, HPV vaccine policies, availability, costs, financial assistance, and education materials vary widely across states or even more defined geographic regions [39]. As a result, variation at state and national levels may not reflect the variation in HPV vaccine uptake occurring at a local level.

Of note, the proportion of all adults in this study who had been vaccinated against HPV (ie, received at least one dose of an HPV vaccine) was 45.3% (66.5% for women and 13.0% for men). These estimates are much higher than the HPV vaccine coverage estimates from the 2012 NHIS for women (34.5%) and men (2.3%) aged 19-26 years (Table 3) [40]. Although the results for women are more similar to those obtained from the NIS-Teen for girls (53.8%), the estimate for men is much lower than the NIS-Teen estimate for boys (20.8%) aged 13-17 years who received at least one dose of HPV vaccine in 2012 [41]. Although the differences in the observed rates may be partially explained by the sampling frame, response rates, or the small number of eligible respondents who received the HPV vaccine question series in the national surveys, the estimates of HPV vaccine uptake are noticeably different from the current study.

**Table 3:** HPV vaccine coverage estimates for men and women in the United States from three surveys.

	HPV vaccine coverage (≥1 dose)			
	Men		Women	
Survey	%	95% CI^a^	%	95% CI
SMASH^b^	13.0	9.4-16.5	65.6	61.6-69.6
NHIS	2.3	1.6-3.4	34.5	31.7-37.3
NIS–Teen	20.8	19.3-22.3	53.8	51.9-55.7

^a^95% confidence interval.

^b^Data are from the Survey of Minnesotans About Screening and HPV, 2013.

### Limitations

Limitations include the fact that the survey responses were self-reported by persons over the Internet and may be subject to under or overreporting. However, other Internet-based studies have shown increased self-disclosure and reporting with online surveys, which may reduce potential response biases (eg, interviewer bias or social desirability) [19,21]. Additionally, there was no failproof method to ensure that survey responses were unique, and there remains a small probability that some participants responded more than once. We also cannot be certain that those that saw the Facebook advertisements were the same people who completed the survey. The 10% of respondents who were not located within the targeted geographical area may be due to the sharing of the study website with friends, or due to outdated user profiles (ie, Facebook thinks a user lives within the study area and displays the ad although the user has since relocated outside of the target area but has not updated their account info), or because the advertisement algorithm was misspecified by Facebook.

### Conclusions

To our knowledge, this is the first study to estimate local level vaccination uptake among young men in the United States. Understanding the local variation and patterns of HPV vaccination of young men could serve to identify areas where HPV infection-related health disparities may continue if neglected. In particular, the online survey also allowed us to collect data on sexual orientation, which in turn would allow us to understand whether men who have sex with men, who are at high risk of HPV-related anal cancer, are receiving the vaccine and to also determine whether reductions in the overall risk of HPV infection will affect transmission to females [42,43].

The results from this study suggest that more detailed and local assessments of HPV vaccine uptake are necessary as estimates vary greatly from national surveys. In addition, recruiting young adults via the Internet is efficient, cost-effective, and can produce a representative sample of the target population. Future work is needed to understand the pattern of HPV vaccine uptake at local levels in order to identify areas that may be best served by vaccine programs.

## Abbreviations

HPV: human papillomavirus
IP: Internet Protocol
NHIS: National Health Interview Survey
NIS: National Immunization Survey
